# Neurofilament light chain (Nf-L) in cerebrospinal fluid and serum as a potential biomarker in the differential diagnosis of neurological diseases in cattle

**DOI:** 10.1186/s13567-024-01441-4

**Published:** 2025-01-10

**Authors:** Giorgia Di Muro, Carlotta Tessarolo, Giulia Cagnotti, Alessandra Favole, Sara Ferrini, Ugo Ala, Claudio Bellino, Giuliano Borriello, Marina Gallo, Giulia Iamone, Barbara Iulini, Marzia Pezzolato, Cristina Casalone, Maria Caramelli, Lorenzo Capucci, Patrizia Cavadini, Cristiano Corona, Antonio D’Angelo

**Affiliations:** 1 Department of Veterinary Sciences, University of Turin, Largo Paolo Braccini 2-5, 10095 Grugliasco, TO Italy; 2 Istituto Zooprofilattico Sperimentale del Piemonte, Liguria e Valle d’Aosta, Via Bologna 148, 10154 Turin, TO Italy; 3 Istituto Zooprofilattico Sperimentale della Lombardia ed Emilia Romagna, Via Bianchi 9, 25124 Brescia, BS Italy

**Keywords:** Bovine, neurology, biomarkers, neurofilament light chain, cerebrospinal fluid

## Abstract

Neurofilament light chain (Nf-L) is a biomarker for axonal damage in human neurology but is understudied in cattle. With this study we wanted to determine Nf-L stability at two different storage temperatures and Nf-L levels in healthy cattle and the relationship with age, evaluate whether Nf-L holds diagnostic potential for neurological disorders, and whether an association exists between Nf-L in serum and in cerebrospinal fluid (CSF). To do this, we measured Nf-L levels in CSF and serum samples from 49 healthy and 75 sick cattle. Storage at −80 °C or −20 °C had no impact on Nf-L concentration. Physiological median Nf-L levels were 6.3 pg/mL (serum) and 414 pg/mL (CSF) in calves and 5.5 pg/mL (serum) and 828 pg/mL (CSF) in adult cattle. There was no association between Nf-L levels in CSF and calf age (r^2^ 0.07, *p* = 0.13), while a weak association was found for Nf-L in serum (r^2^ 0.26, *p* = 0.01), and a significant association in adult cattle (CSF, r^2^ 0.69, *p* = 0.0001; serum, r^2^ 0.68, *p* = 0.0003). CSF Nf-L levels were higher in samples from animals with degenerative (median Nf-L 49971 pg/mL) and infectious central nervous system (CNS) disorders (median Nf-L, age < 2 months 8863 pg/mL; age 2–12 months 17474 pg/mL; age 1–6 years 3546 pg/mL), CNS anomalies and metabolic/toxic disorders. There was a significant association between CSF Nf-L and serum Nf-L in cattle with neurological disorders (r^2^ 0.2, *p* = 0.009). Taken together, these findings suggest the potential of Nf-L as a diagnostic tool in cattle neurology.

**Supplementary Information:**

The online version contains supplementary material available at 10.1186/s13567-024-01441-4.

## Introduction

Neurological disorders in cattle raise considerable problems for beef and dairy cattle farmers and clinicians. They account for approximately 3–8% of cases, and have various origins including infectious agents, nutritional deficiencies, and genetic predispositions [1]. In clinical practice, diagnosing a neurological disorder relies on thorough assessment, including history taking, consideration of signalment, evaluation of clinical signs and their progression, neurological examination, complete blood work, and cerebrospinal fluid (CSF) analysis. Since the root cause of neurological signs is often revealed at necropsy, sensitive, reliable, non-invasive and inexpensive biomarkers are needed that can facilitate diagnosis and treatment, improve the accuracy of differential diagnosis and limit economic losses.

Among all the biomarkers in human and veterinary medicine, neurofilament is drawing growing attention. Neurofilaments are major components of the axonal cytoskeleton [2] and consist of three highly conserved subunits across human and other animal species: Nf-light (Nf-L; 68–86 kDa), Nf-medium (145–160 kDa), and Nf-heavy (200–220 kDa) chains [3]. Following axonal damage due to degeneration, traumatic or vascular injury, neoplasia or inflammation, Nf-L, the most soluble subunit, is released into the extracellular space and then into the CSF and bloodstream where it can be measured [4, 5].

In human medicine, Nf-L is widely accepted as a marker for axonal damage, disease activity and progression or treatment response in neurological diseases such as Alzheimer’s and Parkinson’s diseases, amyotrophic lateral sclerosis, multiple sclerosis, Creutzfeldt-Jakob disease, inherited and acquired peripheral neuropathies, traumatic brain injury and bacterial meningitis [6–12]. Furthermore, studies have reported an age-related increase of Nf-L in CSF, serum, and plasma in healthy subjects [13–16].

Since the molecular signatures of many proteins are highly conserved among species, Nf-L could be a useful marker in the differential diagnosis of diseases in animals. However, little is known about Nf- L levels in healthy animals or its potential as a biomarker in neurological disorders. Studies reported that the Nf-L concentration in dogs mirrors the dynamics of Nf-L in humans: it is detectable in serum, plasma, and CSF and its concentration increases with age [17]. Furthermore, in dogs with central nervous system (CNS) disorders, Nf-L concentration was found similar to that in humans [3, 4, 17–22]. In their study on sheep, Zetterberg and colleagues measured Nf-L levels in the diagnosis of prion diseases [23]. Two studies reported the use of Nf-L measurement in the diagnosis of traumatic brain injury in experimental swine models [24, 25]. Mengel and colleagues investigated its association with disease progression of spinocerebellar ataxia type 3 in a mouse model [26], while more recently, Perego et al. measured Nf-L concentration in rat with hypoxic ischemic brain injury following cardiac arrest [27].

To our best knowledge, there are no reports describing Nf-L levels in healthy and sick cattle. To fill this gap, we measured Nf-L concentration in serum and CSF samples collected from cattle presenting neurological symptoms of various origins and from healthy controls. Here we chose the Ella assay (ProteinSimple, part of Bio-Techne, Minneapolis, MN, USA) from among the variety of techniques commercially available for Nf-L quantification, as discussed by Kuhle et al. [28] and Truffi et al. [29]. The Ella assay was the preferred option because inexpensive and user friendly. It works with a microfluidic cartridge-based immunoassay platform widely used to quantify in triplicate soluble biomarkers such as cytokines, chemokines, and Nf-L and can measure 72 samples in a single run.

The four aims of the present study were: determine the Nf-L stability in bovine CSF and serum samples stored for an extended period at two different temperatures; define CSF and serum Nf-L concentrations in healthy cattle and the possible relationship with animal age; compare CSF Nf-L levels between healthy cattle and cattle with neurological disorders to determine the potential of Nf-L as a biomarker for neurological diseases; explore the possible relationship between CSF and serum Nf-L measurements to determine whether serum Nf-L could serve as a less invasive proxy measure of CSF Nf-L.

## Materials and methods

### Ethical statement

This study adhered to current animal welfare regulations (Directive 98/58/EC and Italian Decree Law 146/2001). All procedures were conducted as described by the institutional guidelines in accordance with national (D.L.26/2014) and international laws and policies (EEC Council Directive 63/2010). Ethical approval for the study was obtained from the Bioethics Committee of the University of Turin (protocol no. 0251347) and from the Italian Ministry of Health (Auth. no. 242/2020–PR). Owners provided written informed consent for veterinary assessment and treatment of their animals.

The study population was healthy cattle and sick cattle. Samples were collected during routine analysis for diagnostic procedures.

#### Healthy animal group

A healthy control group was prospectively enrolled from January 2021 to December 2023 and consisted of cattle judged healthy based on unremarkable general and neurological examination and normal blood and CSF analysis.

#### Sick animal group

Patients referred to the Neurology Service of the Veterinary Teaching Hospital (VTH), University of Turin, from January 2021 to December 2023, for clinical signs suggestive of a neurological disorder were prospectively recruited. The medical records of patients with neurological conditions referred to the VTH between July 2017 and December 2020, and whose CSF samples had been stored at −20 °C, were retrospectively reviewed.

### Inclusion and exclusion criteria

For each animal, signalment and medical history were entered in a database. All animals underwent a complete clinical and neurological evaluation (inspection of their mental state, behavior, gait with assessment of proprioception and postural reactions, cranial nerves and spinal reflexes) by a board-certified neurologist (ADA). Furthermore, complete blood and CSF analyses were performed for each animal. Only patients for whom a definitive diagnosis was obtained through the aforementioned investigations, response to the pharmacological treatment, and/or postmortem examination were included in the study. Sick animals were grouped according to the VITAMIN D acronym (Vascular, Infectious/inflammatory, Trauma, Anomaly, Metabolic/toxic, Idiopathic, Neoplasia, Degenerative) [30].

### CSF and serum samples

Blood was sampled at the coccygeal vein or jugular vein with 20-gauge needles. Blood samples were stored both in tubes containing anticoagulant (EDTA), for the haemochromocytometric profile, and in tubes with coagulation activator with serum separator for evaluation of a complete biochemical profile including electrolytes. The samples were stored at 4 °C and all hematological investigations were performed within 1 h of blood sampling at the VTH analytical laboratory. Serum samples were separated by centrifugation at 3000 *g* for 10 min at 4 °C, aliquoted into 2 mL tubes, and stored both at −80 °C and −20 °C for Nf-L quantification and further analysis.

CSF was sampled at the lumbosacral level with the animal either stationary or in sternal decubitus, depending on its ability to remain stationary. The sampling site (5 × 10 cm) was shaved and locally anesthetized with 2.5 mL of procaine hydrochloride 20 mg/mL (Procamidor, Richter Pharma Ag, Wels, Austria). The area around the sampling site was disinfected by applying povidone iodine and alcohol-moistened gauze alternately three times. CSF was obtained using sterile spinal 18-gauge needles (adults) or 21-gauge needles (calves), collected with 5 mL sterile syringes, and stored in plain tubes at 4 °C. The CSF samples were aliquoted in 2 mL tubes. One part underwent within 1 h physicochemical analysis (total and differential nucleated cells count, total red blood cells count, microprotein concentration) at the VTH analytical laboratory, while the other part was stored both at −80 °C and −20 °C for Nf-L quantification and further analysis.

### Nf-L measurement in CSF and serum samples

The CSF and serum samples were gently thawed and centrifuged at 3000 *g* for 10 min at 4 °C before analysis. Since Nf-L is a highly conserved protein and no species-specific assays are commercially available, we ran the Human NF-L Simple Plex assay (ProteinSimple, San Jose, CA, USA) on the Ella device (ProteinSimple), according to the manufacturer’s instructions. This instrument is characterized by a validated lack of cross-reactivity with similar proteins and has a limit of detection (LOD) for Nf-L of 1.09 pg/mL, with a sample recovery rate of 100% (the main competitor on the market has a recovery rate of 68%) [31]. The Ella device was calibrated using the in-cartridge factory standard curve. Serum and CSF samples were measured in a 1:2 dilution in Sample Diluent (ProteinSimple). A single well was used for each sample since triplicates assays are automatically performed on the Simple Plex assay microfluidic platform. The lower limit of quantification was 2.70 pg/mL; the upper limit was 10 290.00 pg/mL.

### Grouping and statistical analysis

Statistical analysis was performed using the Prism 10.2.0.392 software (GraphPad software LLC, Boston, MA, USA). Normal distribution of data was tested using the Shapiro–Wilk test. Since not all quantitative variables had normal distribution, their descriptive statistics are expressed as median and interquartile range (IQR), with nonparametric tests for analysis. Categorical variables are expressed as absolute frequency and percentage.

#### Nf-L stability at two different storage temperatures

Differences in Nf-L concentration measured in duplicate samples of CSF and serum stored both at −20 °C and −80 °C were assessed with the Wilcoxon signed-rank test and correlation was tested with Spearman’s rank correlation test.

#### Nf-L concentrations in healthy cattle

We wanted to determine whether a relationship exists between serum and CSF Nf-L levels and animal age. Drawing on evidence from previous studies involving healthy dogs, children, and adult humans, we divided the healthy cattle into two age-based subgroups: calves (< 12 months) and adults (≥ 1 year). The subgroups underwent separate analysis with Spearman’s rank correlation test and a simple linear regression. Nf-L levels were log_10_-transformed for regression analysis and the results are reported as r squared and *p*-value of the F test.

Since age is known to play a role in susceptibility to diseases, the healthy cattle were further grouped by age into five subgroups (< 2 months, ≥ 2–12 months, ≥ 1–6 years, ≥ 6–12 years, ≥ 12 years). The Kruskall-Wallis test and subsequent pairwise comparisons were used to assess differences in Nf-L concentration between the age groups; the Benjamini–Hochberg procedure was applied to adjust the *p*-values.

#### Comparison of CSF Nf-L concentration in healthy and sick cattle

Sick cattle categorized according to the VITAMIN D acronym were further subdivided into the same five age-based groups as the healthy animals. CSF Nf-L concentration in healthy and sick cattle matched for age was compared using the Mann–Whitney test. Furthermore, to determine whether the various disorders could be differentiated based on Nf-L concentration, the CSF Nf-L concentration across the VITAMIN D groups matched for age was compared. The Kruskal–Wallis test and subsequent pairwise comparisons were employed to compare sick animals less than 2 months old, as more than two classes of VITAMIN D were identified in these animals; the Benjamini–Hochberg procedure was applied to adjust the *p*-values. The Mann–Whitney test was applied to animals of 2–12 months of age.

The area under the ROC curve (AUROC) was calculated to identify the optimal cut-off value of CSF Nf-L concentrations that could distinguish between healthy and sick animals, as well as differentiate between the diseases. The Youden index was employed to determine the optimal cut-off point.

#### Relationship between serum and CSF Nf-L concentration

Simple linear regression analysis was used to assess the relationship between Nf-L concentrations in serum and in CSF samples from healthy and sick cattle. The Nf-L levels were log_10_-transformed for regression analysis and the results are reported as r squared and p-value of the F test.

A *p*-value < 0.05 was considered statistically significant for all statistical tests.

## Results

### Study sample

The study sample was 49 healthy cattle and 75 with neurological disorders (46 included prospectively and 29 included retrospectively). A total of 124 CSF samples were collected, 49 from healthy animals and 75 from sick animals, plus a total of 71 serum samples, 39 from healthy animals and 32 from sick animals. Additional file 1 presents the demographics of the healthy animals; Additional file 2 presents the demographics of the sick cattle.

### Nf-L stability at two different storage temperatures

**Figure 1:**
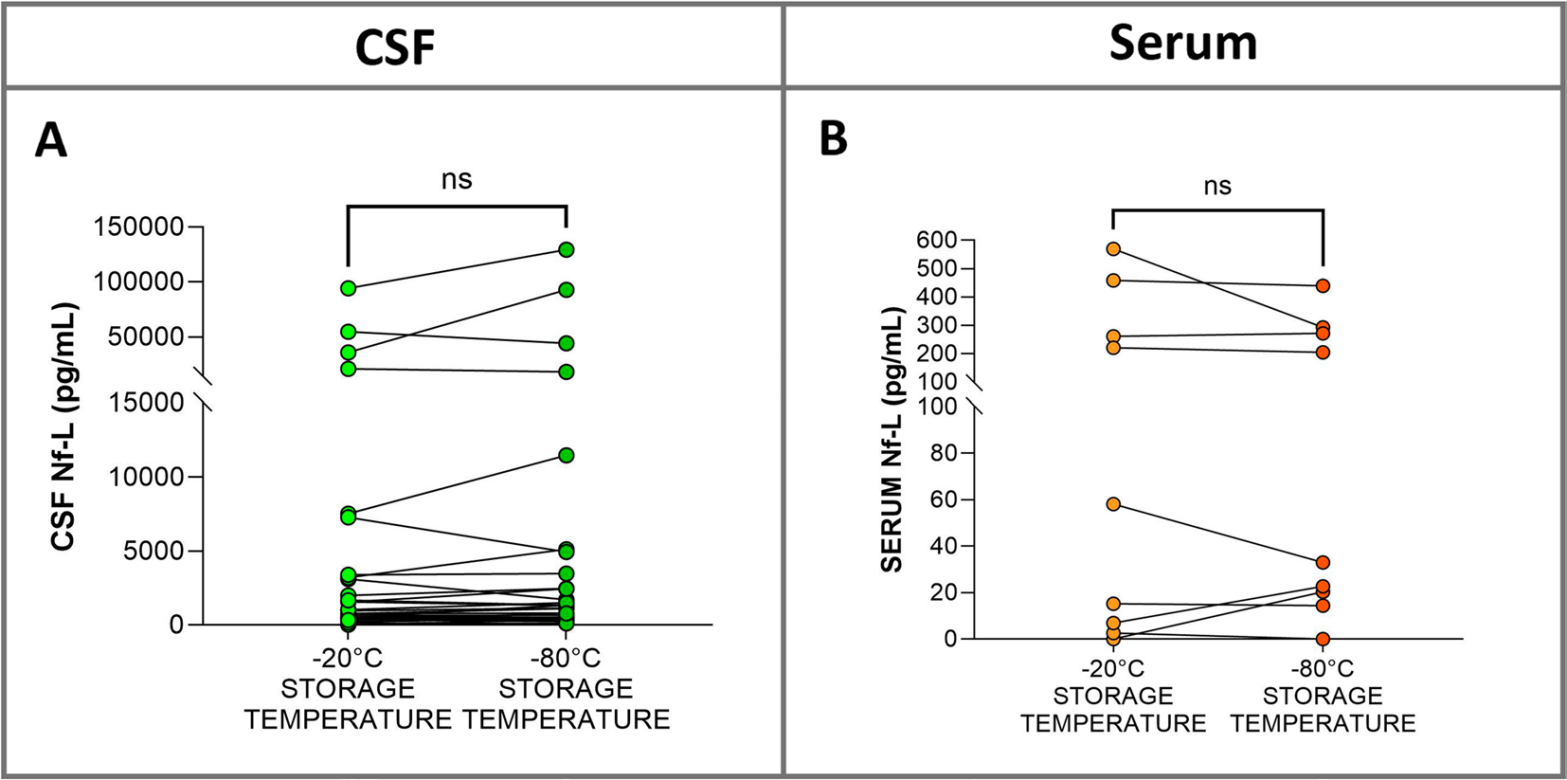
**Nf-L stability at different storage temperatures.** There were no substantial differences in Nf-L levels between CSF (**A**) and serum (**B**) samples stored at −20 °C and at −80 °C. Wilcoxon matched-pairs signed rank test was used. CSF denotes cerebrospinal fluid, Nf-L neurofilament light chain, IQR interquartile range, ns not significant.

**Figure 2:**
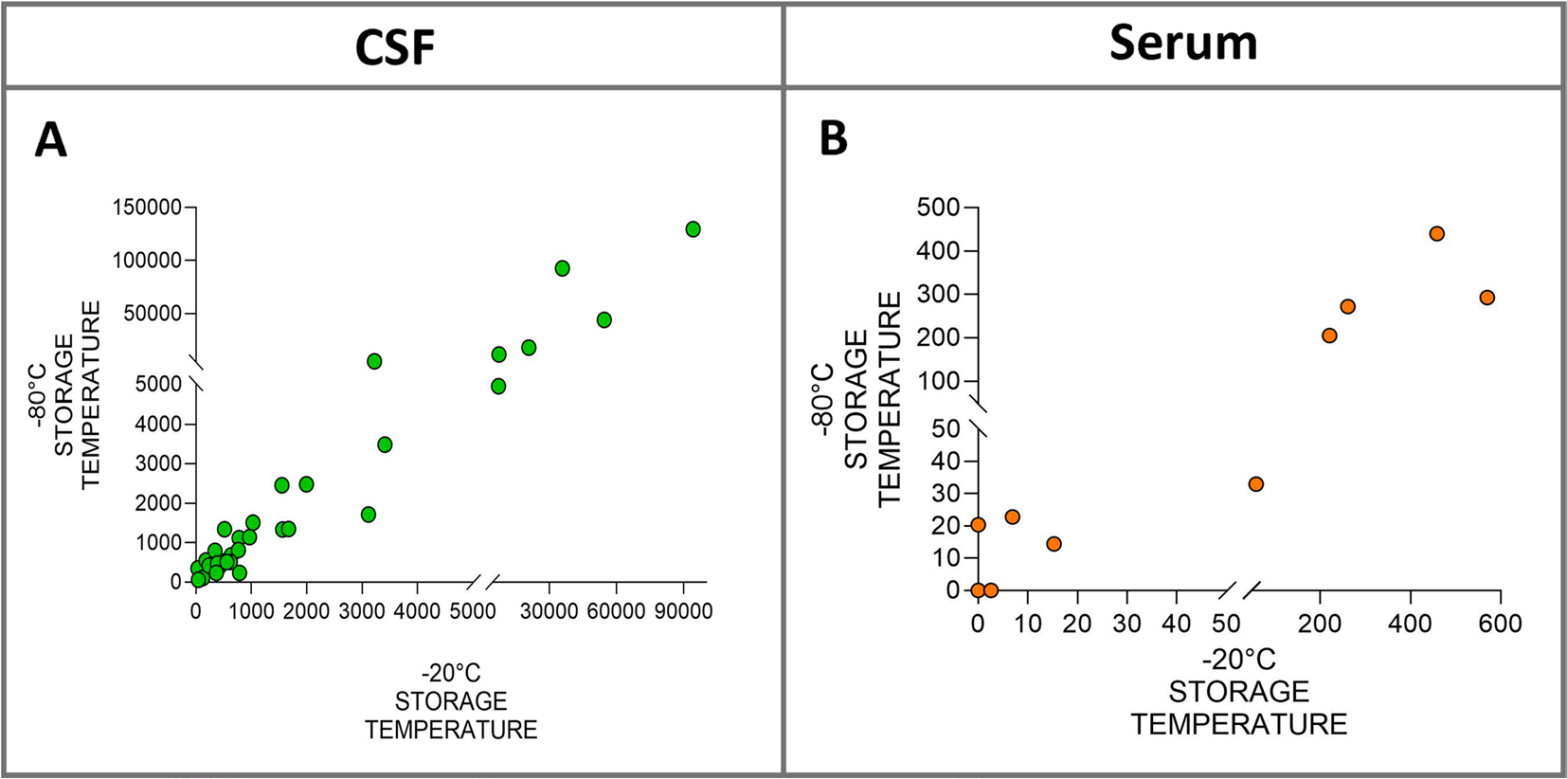
**Correlation analysis of Nf-L levels in CSF and serum stored at −20 °C and at −80 °C.** Spearman’s rank correlation test showed a positive correlation between Nf-L levels in CSF (**A**) and serum (**B**) samples stored in duplicate at −20 °C and −80 °C. The scatter points denote individual data points. CSF denotes cerebrospinal fluid, Nf-L neurofilament light chain.

To determine whether storage temperature could affect Nf-L stability, the CSF and serum samples from sick animals stored at −80 °C were compared with those stored at −20 °C. The samples were stored prior to analysis for a median duration of 16.5 months, (IQR, 9–20 months). There was no significant difference in Nf-L concentration between the samples stored at −80 °C and those stored at −20 °C (CSF, *p* = 0.19, Figure 1A; serum, *p* = 0.34; Figure 1B). Correlation of Nf-L concentration measured in the samples stored in duplicate at the two temperatures was also tested. The results showed a strong positive correlation for CSF (rho = 0.91, *p* < 0.0001, Figure 2A) and serum (rho = 0.94, *p* < 0.0001, Figure 2B), suggesting the viability of samples stored at −20 °C for analysis.

### Nf-L concentration in healthy cattle

**Figure 3:**
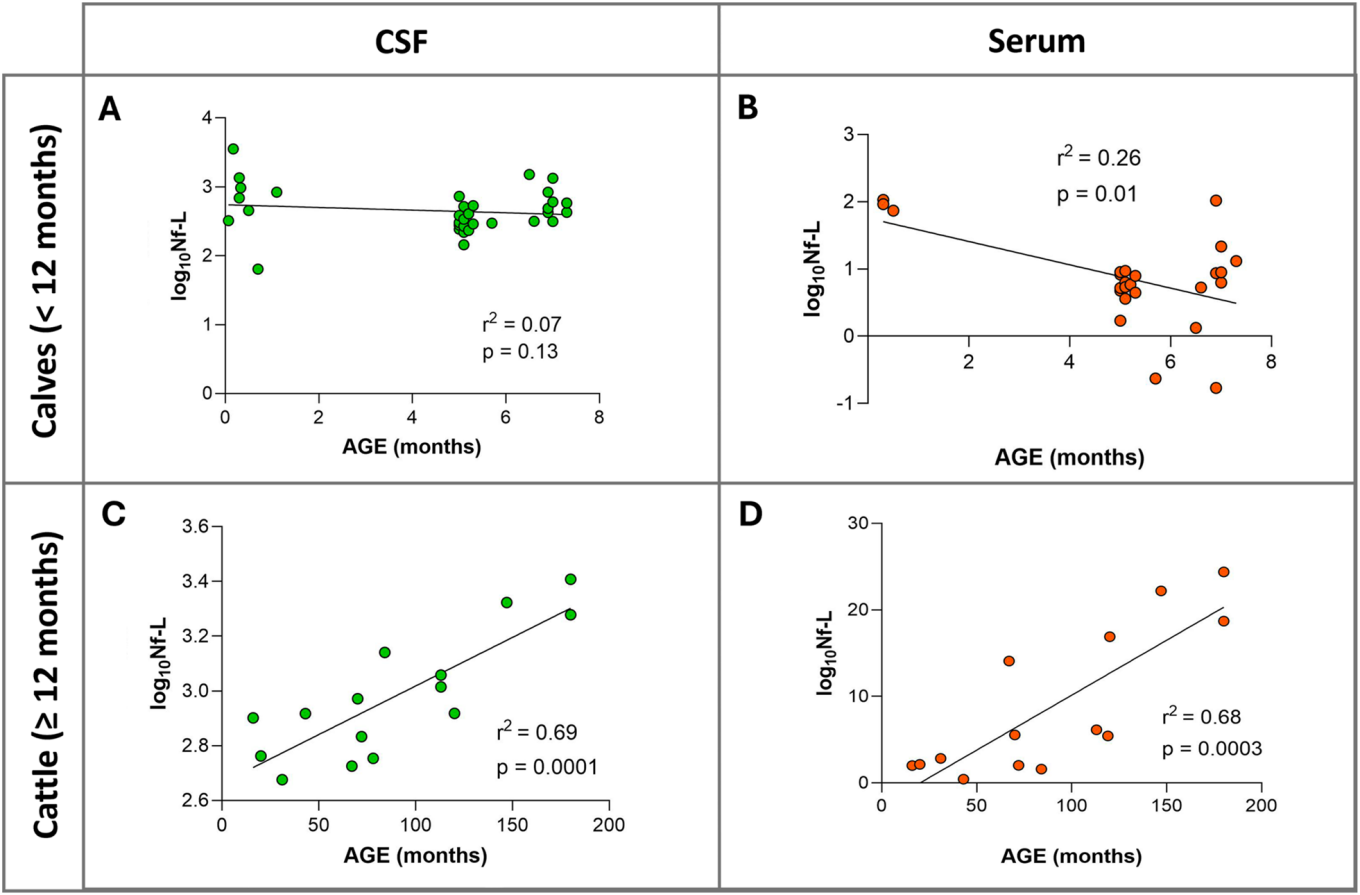
**Relationship between age and serum (A and C) and CSF (B and D) Nf-L levels in healthy animals.** There was a weak relationship between animal age and serum Nf-L in samples from calves (**A**). There was a positive association between serum Nf-L and age in samples from adults (**C**). There was no relationship between age and CSF Nf-L levels in calves (**B**). There was a positive association between CSF Nf-L levels and age in adults (**D**). The simple linear regression goodness of fit is expressed as r squared (r^2^) and related *p*-value. The scatter points denote data points. CSF denotes cerebrospinal fluid, Nf-L neurofilament light chain.

Correlation between serum and CSF Nf-L concentrations and age was tested in calves (< 12 months, *n* = 34) and in adult cattle (≥ 1 year, *n* = 15). The median age was 5.1 (5–6.6) months for the calves and 78 (55.2–120) months for the adults. The median Nf-L concentration was 6.3 (4.8–9.4) pg/mL in serum and 414 (290.8–665.5) pg/mL in CSF in the calves and 5.5 (2.1–16.2) pg/mL in serum and 828 (630–1262.5) pg/mL in CSF in the adults. No correlation was found between age and Nf-L concentration in either CSF (rho = −0.005, *p* = 0.98) or serum (rho = −0.09, *p* = 0.7) in the calves. Differently, there was a positive correlation between CSF and serum Nf-L levels and age (CSF, rho = 0.79, *p* = 0.0008; serum, rho = 0.74, *p* = 0.003) in the adults. Simple linear regression conducted separately for the two groups to determine a possible predictive role of age for Nf-L levels revealed no relationship between age and CSF Nf-L levels (r squared = 0.07, *p* = 0.13, Figure 3A) and a weak predictive role of age for serum Nf-L levels (r squared = 0.26, *p* = 0.01, Figure 3B) in the calves. Differently, there was a moderate relationship between age and CSF Nf-L concentration (r squared = 0.69, *p* = 0.0001, Figure 3C) and serum Nf-L concentration (r squared = 0.68, *p* = 0.0003, Figure 3D) in the adults.

**Figure 4:**
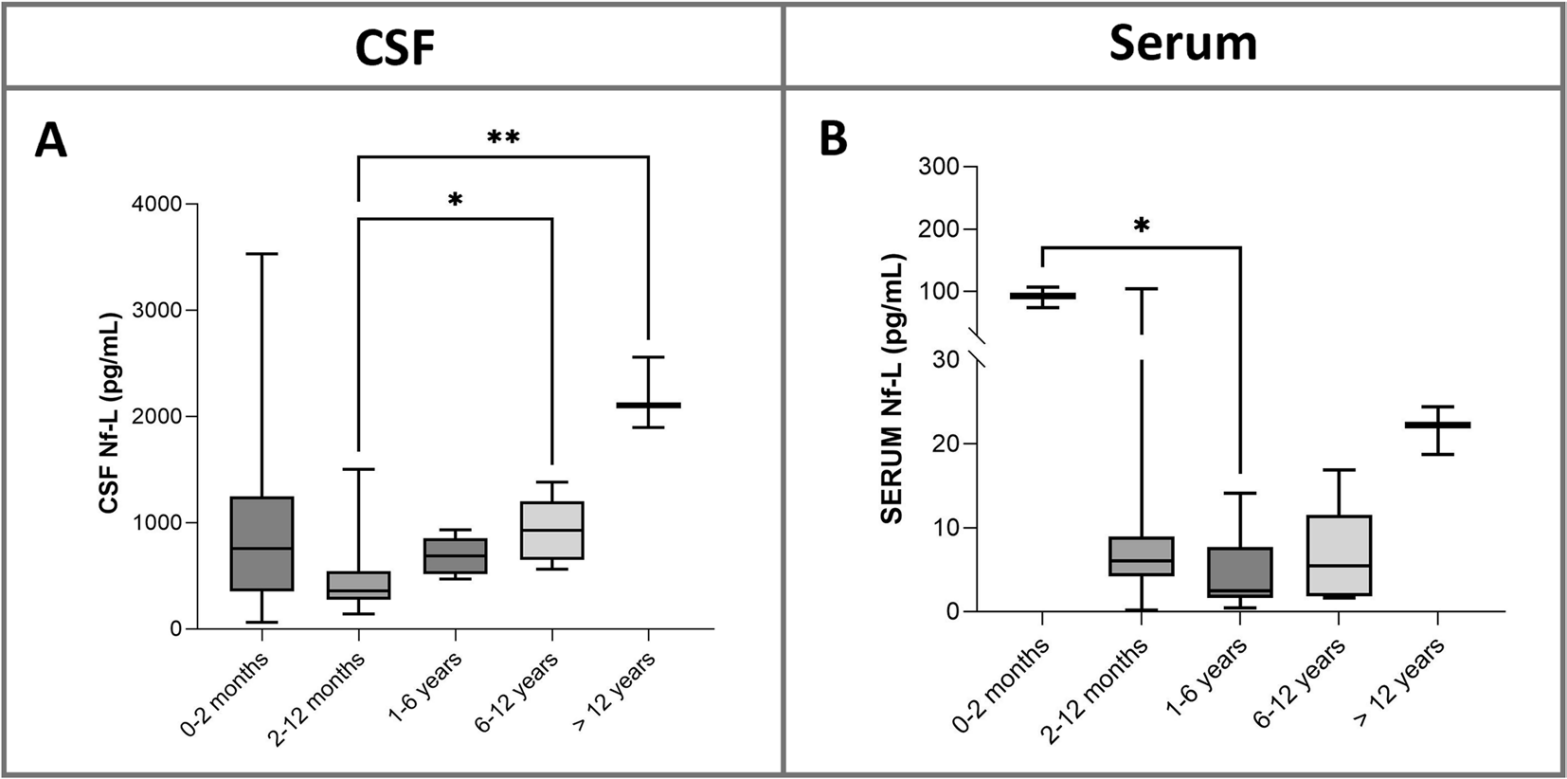
**Comparison between CSF (A) and serum (B) Nf-L levels across age groups of healthy animals.** There were significant differences in CSF NF-L levels between animals aged 2–12 months and 6–12 years, and between animals aged 2–12 months and ≥ 12 years (**A**), and in serum NF-L levels between animals aged 0–2 months and 1–6 years (**B**). The median, IQR, maximum and minimum are shown. The Kruskall-Wallis test with pairwise comparisons was used, and the Benjamini–Hochberg procedure applied to adjust the *p*-values. Asterisks (*) denote the level of statistical significance: *p < 0.05, **p < 0.01. CSF denotes cerebrospinal fluid, IQR interquartile range, Nf-L neurofilament light chain.

Comparison of serum and CSF Nf-L concentrations across age groups showed significantly higher CSF Nf-L levels in the group aged 2–12 months than in the group aged 6–12 years (*p* = 0.04) and in the group aged ≥ 12 years compared with the group aged 2–12 months (*p* = 0.007; Figure 4A). Serum Nf-L levels were significantly higher in the group aged 0–2 months than in the group aged 1–6 years (*p* = 0.02; Figure 4B). No significant differences were found for the other groups.

### Comparison of CSF Nf-L concentration in healthy and sick cattle

Additional files 3 and 4 present the CSF Nf-L concentration in the healthy and the sick animals across different age groups, respectively.

#### Healthy vs calves with anomalies (age, < 2 months)

**Figure 5:**
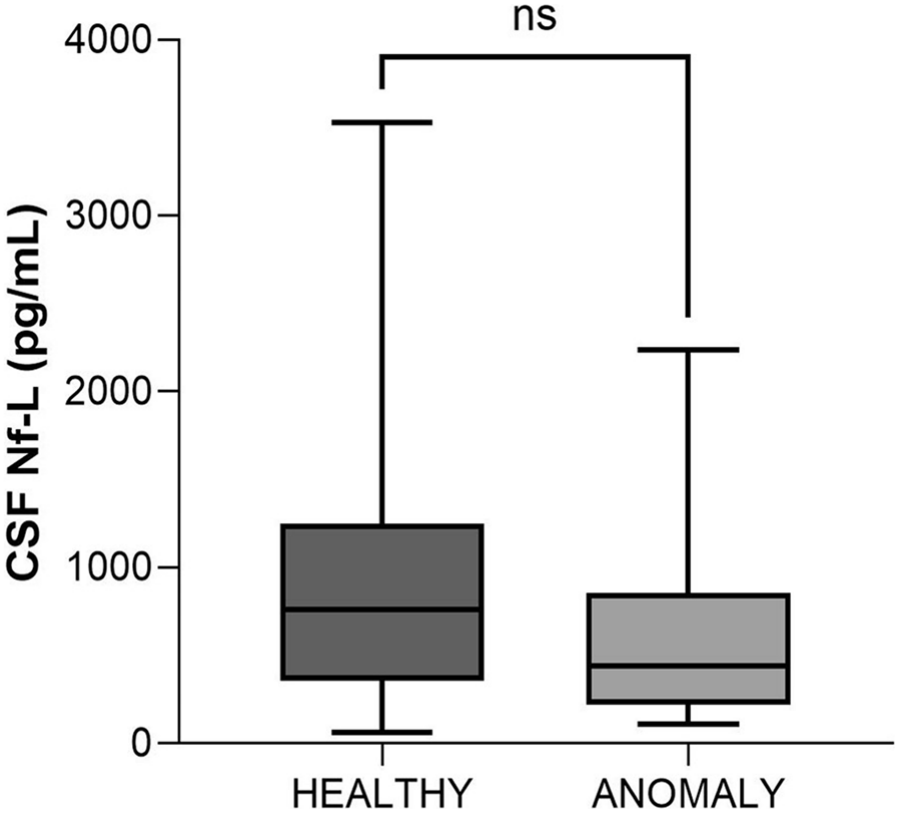
**Comparison of CSF Nf-L levels between healthy animals and calves (age, < 2 months) with CNS anomaly.** There were no substantial differences in CSF Nf-L levels between healthy calves and those with CNS anomalies. The median, IQR, maximum and minimum are shown. The Mann–Whitney test was used. ns denotes not significant. CSF denotes cerebrospinal fluid, Nf-L neurofilament light chain, CNS central nervous system, IQR interquartile range.

The anomalies in these calves included cerebellar hypoplasia (*n* = 5), syringohydromyelia (*n* = 4, 2 with cervical and cervico-thoracic spinal cord, 1 with the thoraco-lumbar spinal cord and 1 with the lumbo-sacral spinal cord), congenital hydrocephalus (*n* = 3), and spina bifida and myelomeningocele (thoraco-lumbar spinal cord) (*n* = 1) [32]. There was no significant difference in CSF Nf-L concentrations between healthy calves and those with CNS anomalies (*p* = 0.34, Figure 5).

#### Healthy vs calves with degenerative disorders (age, < 2 months)

**Figure 6:**
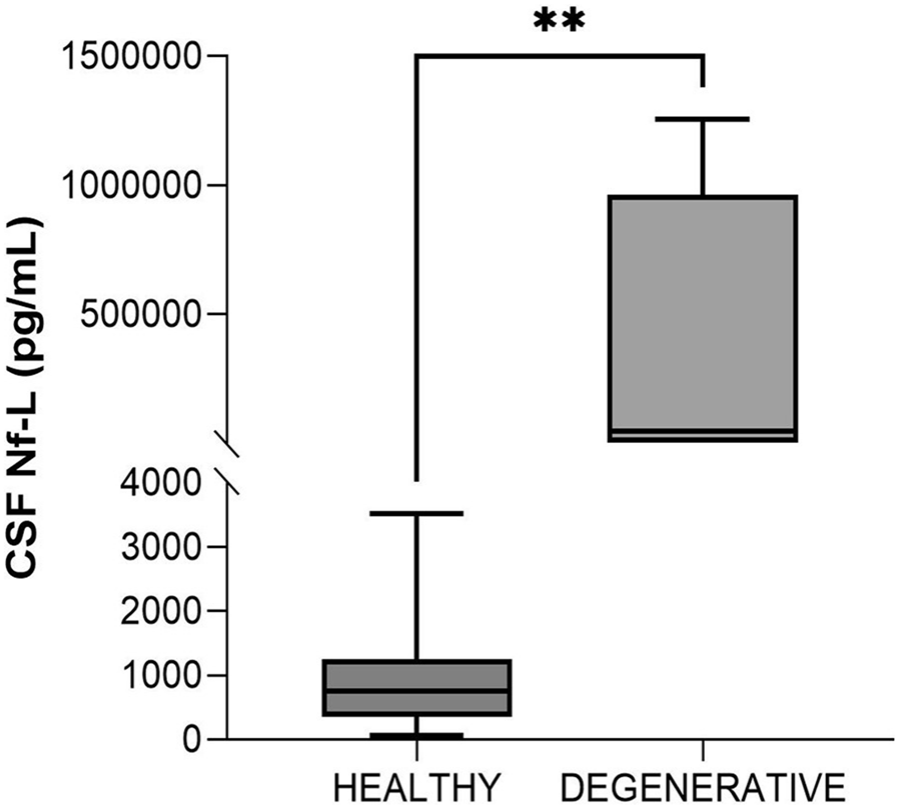
**Comparison of CSF Nf-L levels between healthy animals and calves (age, < 2 months) with CNS degenerative disorders.** CSF Nf-L levels in calves with a neurodegenerative disorder were significantly higher than those in healthy calves. The median, IQR, maximum and minimum are shown. The Mann–Whitney test was used. Asterisks (*) denote the level of statistical significance: **p < 0.01. CSF denotes cerebrospinal fluid, CNS central nervous system, IQR interquartile range, Nf-L neurofilament light chain.

**Figure 7:**
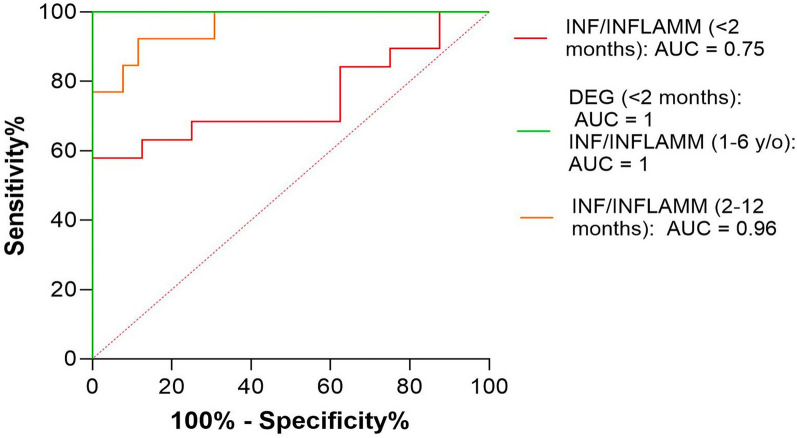
**AUROC curves predicting CNS disorder based on CSF Nf-L concentration.** The AUROC curve for CSF Nf-L concentration was 0.75 in calves aged < 2 months with infection/inflammation (red line), 0.96 in calves aged 2–12 months (orange line), and 1 in adults aged 1–6 years (green line). The AUROC curve for CSF Nf-L concentration was 1 in calves with a neurodegenerative disorder (green line). The optimal cut-off for differentiating between healthy and sick animals was 1137 pg/mL (Se, 68.42%, Sp, 75%), 736.5 pg/mL (Se, 92.31%, Sp, 88.46%), 1108 pg/mL (Se, 100%, Sp, 100%), and 4141 pg/mL (Se, 100%, Sp, 100%), respectively. The x-axis represents the false positive rate (1-Specificity), and the y-axis represents the true positive rate (Sensitivity). AUROC denotes area under the receiver operating characteristic curve, CSF cerebrospinal fluid, CNS central nervous system, DEG degenerative, INF/INFLAMM infectious/inflammatory, Nf-L neurofilament light chain, Se sensitivity, Sp specificity.

All the calves in this group were diagnosed with spinal muscular atrophy (SMA) [33]. The CSF Nf-L levels were significantly higher than those in the healthy calves (*p* = 0.004, Figure 6). The AUROC for CSF Nf-L concentration was 1 (Figure 7). The corresponding optimal cut-off values to distinguish between healthy calves and those with SMA was 4141 pg/mL; sensitivity and specificity were 100 and 100%, respectively.

#### Healthy vs calves with infectious/inflammatory disorders (age, < 2 months)

**Figure 8:**
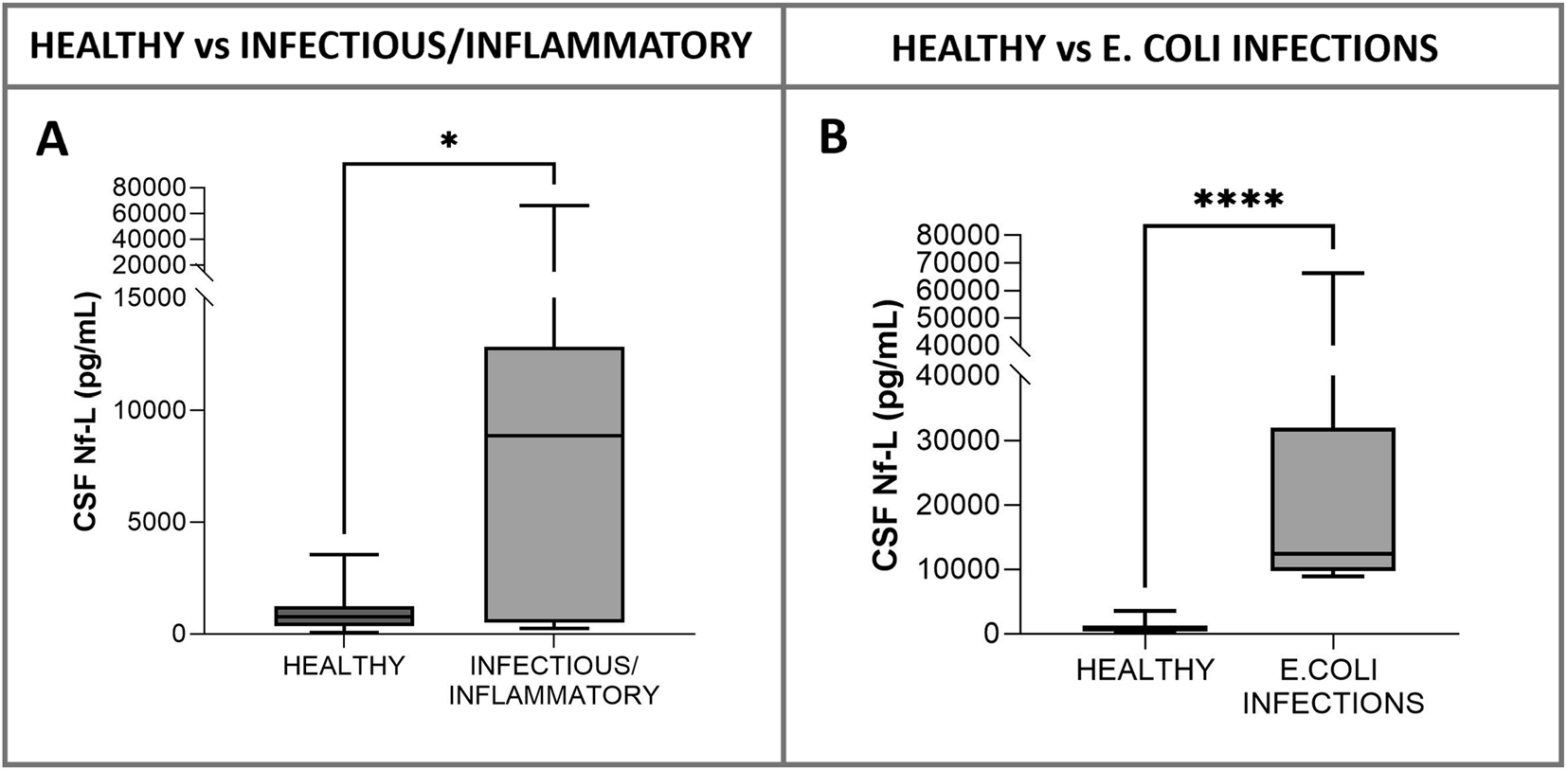
**Comparison of CSF Nf-L levels between healthy calves and those (age, < 2 months) with a CNS infectious/inflammatory disorder (A) and** ***E. coli*** **CNS infection (B)**. CSF Nf-L levels were significantly higher in calves with an infectious/inflammatory disorder (**A**). CSF Nf-L levels were significantly higher in calves with an *E. coli* infection (**B**). The median, IQR, maximum and minimum are shown. The Mann–Whitney test was used. Asterisks (*) denote the level of statistical significance: * p < 0.05, **** p < 0.0001. CSF denotes cerebrospinal fluid, CNS central nervous system, IQR interquartile range, Nf-L neurofilament light chain.

Neonatal meningitis-meningoencephalitis(-myelitis) of suspected bacterial origin was diagnosed antemortem in 2 calves. A bacterial meningitis-meningoencephalitis (-myelitis) was confirmed at necropsy after euthanasia due to deterioration of their neurological and clinical signs. The causative pathogen was identified by the culture of brain samples in 10 of these animals (meningoencephalitis caused by *Escherichia coli* in 9 and meningoencephalitis caused by *Streptococcus uberis* in 1). CSF Nf-L concentrations were significantly higher in the sick calves (*p* = 0.04, Figure 8A). The AUROC for CSF Nf-L concentration was 0.75 (Figure 7). The corresponding optimal cut-off to distinguish between healthy calves and those with CNS infections of suspected bacterial origin was 1137 pg/mL; sensitivity and specificity were 68.42% and 75%, respectively.

Analysis of the subgroup in which *E. coli* meningoencephalitis was identified (CSF Nf-L concentration, 12360 [9778–32046] pg/mL), showed a strong significant difference compared with the healthy calves (*p* < 0.0001, Figure 8B).

#### Healthy vs calves with infectious/inflammatory disorders (age, 2–12 months)

**Figure 9:**
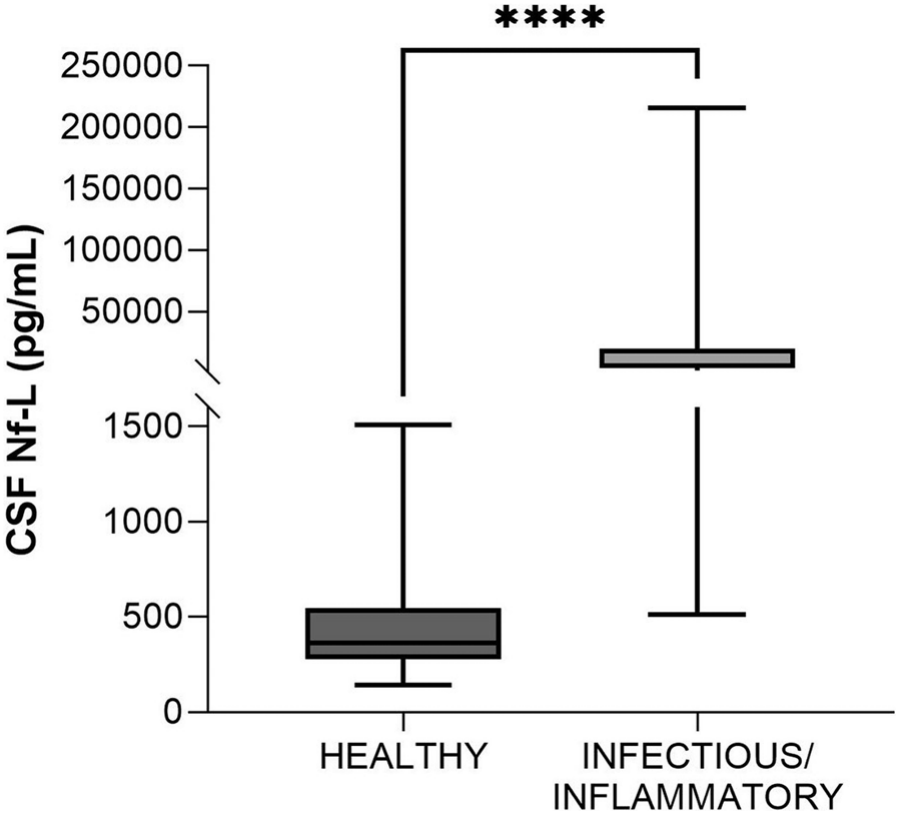
**Comparison of CSF Nf-L levels between healthy calves and those (age, 2–12 months) with a CNS infectious/inflammatory disorder.** CSF Nf-L levels were significantly higher in calves with an infectious/inflammatory disorder. The median, IQR, maximum and minimum are shown. The Mann–Whitney test was applied. Asterisks (*) denote the level of statistical significance: **** p < 0.0001. CSF denotes cerebrospinal fluid, CNS central nervous system, IQR interquartile range, Nf-L neurofilament light chain.

This group comprised 13 cattle, 12 of which with meningitis-meningoencephalitis (-myelitis) of suspected bacterial origin and 1 with meningoencephalitis of suspected yeast origin were enrolled in this group. CSF Nf-L concentrations were significantly higher in the samples from the sick animals (*p* = 0.0001, Figure 9). The AUROC for CSF Nf-L concentration was 0.96 (Figure 7). The corresponding optimal cut-off to distinguish between healthy and sick calves was 736.5 pg/mL; sensitivity and specificity were 92.31 and 88.46%, respectively.

#### Healthy vs adults with infectious/inflammatory disorders (age, 1–6 years)

**Figure 10:**
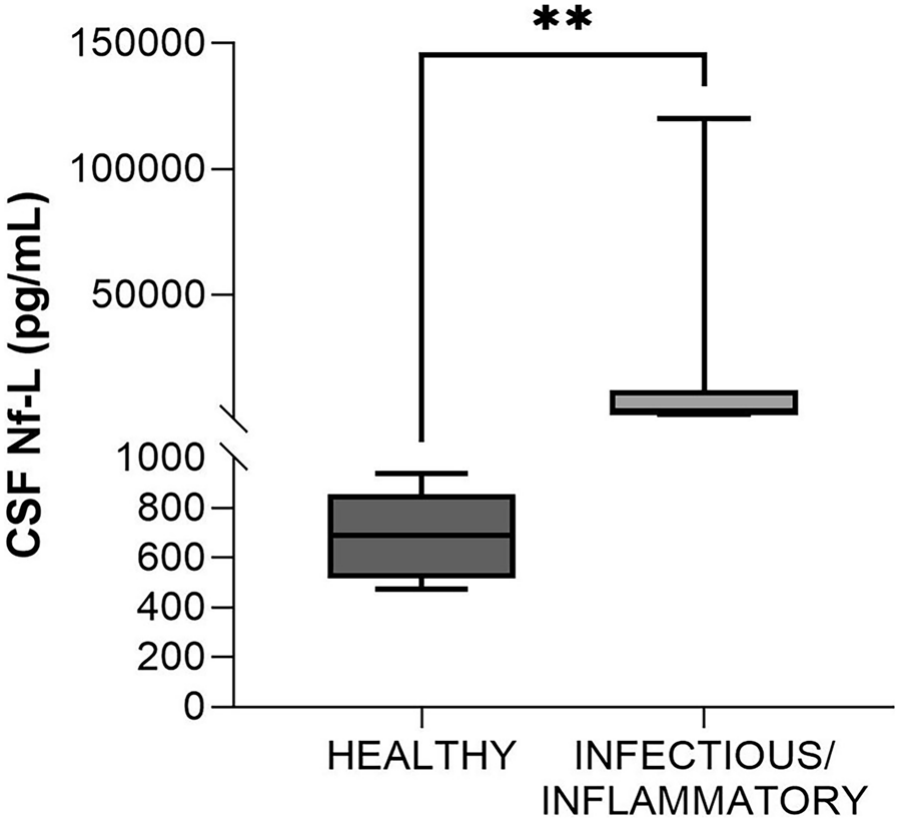
**Comparison of CSF Nf-L levels between healthy cattle and those (age, 1–6 years) with a CNS infectious/inflammatory disorder. **CSF Nf-L levels were significantly higher in cattle with an infectious/inflammatory disorder. The median, IQR, maximum and minimum are shown. The Mann–Whitney test was applied. Asterisks (*) denote the level of statistical significance: **p < 0.01. CSF denotes cerebrospinal fluid, CNS central nervous system, IQR interquartile range, Nf-L neurofilament light chain.

This group comprised 7 cases: suspected *Listeria spp.*-related meningoencephalitis (*n* = 3), meningitis-meningoencephalitis by other not-identified bacteria (*n* = 3), and confirmed bacterial meningoencephalitis caused by *Streptococcus uberis* (n = 1). CSF Nf-L levels were higher in the samples from the sick animals (*p* = 0.001, Figure 10). The AUROC for CSF Nf-L concentration was 1 (Figure 7). The corresponding optimal cut-off to distinguish between healthy cattle and those with CNS infections of suspected bacterial origin was 1108 pg/mL; sensitivity and specificity were 100 and 100%, respectively.

#### Healthy vs calves with metabolic/toxic disorders (age, 2–12 months)

**Figure 11:**
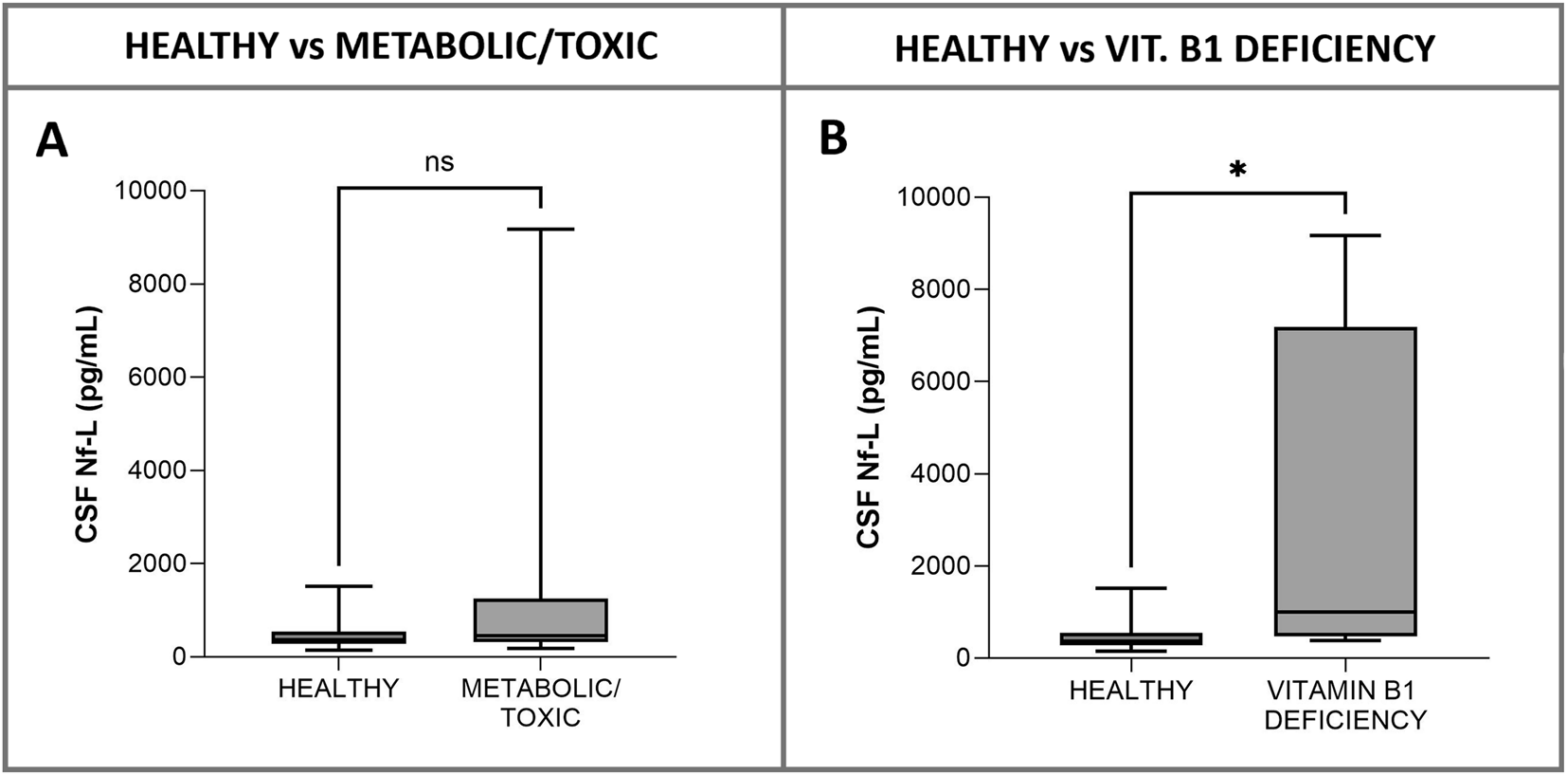
**Comparison of CSF Nf-L levels between healthy cattle and those (age, 2–12 months) with a CNS metabolic/toxic disorder (A) and vitamin B1 deficiency (B).** (**A**) There was no substantial difference in CSF Nf-L concentration between healthy calves and those with a metabolic/toxic disorder. **B** CSF Nf-L levels were significantly higher in calves with a vitamin B1 deficiency. The median, IQR, maximum and minimum are shown. The Mann–Whitney test was applied. Asterisks (*) denote the level of statistical significance: * *p* < 0.05. ns denotes not significant. CSF cerebrospinal fluid, CNS central nervous system, IQR interquartile range, Nf-L neurofilament light chain

Metabolic/toxic disorders were diagnosed in 15 cases: vitamin A deficiency (*n* = 6), calcium/magnesium deficiency (*n* = 5), and vitamin B1 (thiamine) deficiency (*n* = 4). There was no significant difference in CSF Nf-L concentrations between the sick and the healthy calves (*p* = 0.13, Figure 11A). However, when the samples from the sick calves were compared with those from the healthy calves separately for each disorder, the CSF Nf-L levels were significantly higher in the cases of vitamin B1 deficiency (CSF Nf-L levels, 997 [473.3–7194] pg/mL; *p* = 0.04, Figure 11B).

#### Healthy vs adults with metabolic/toxic disorders (age, ≥ 12 years)

**Figure 12:**
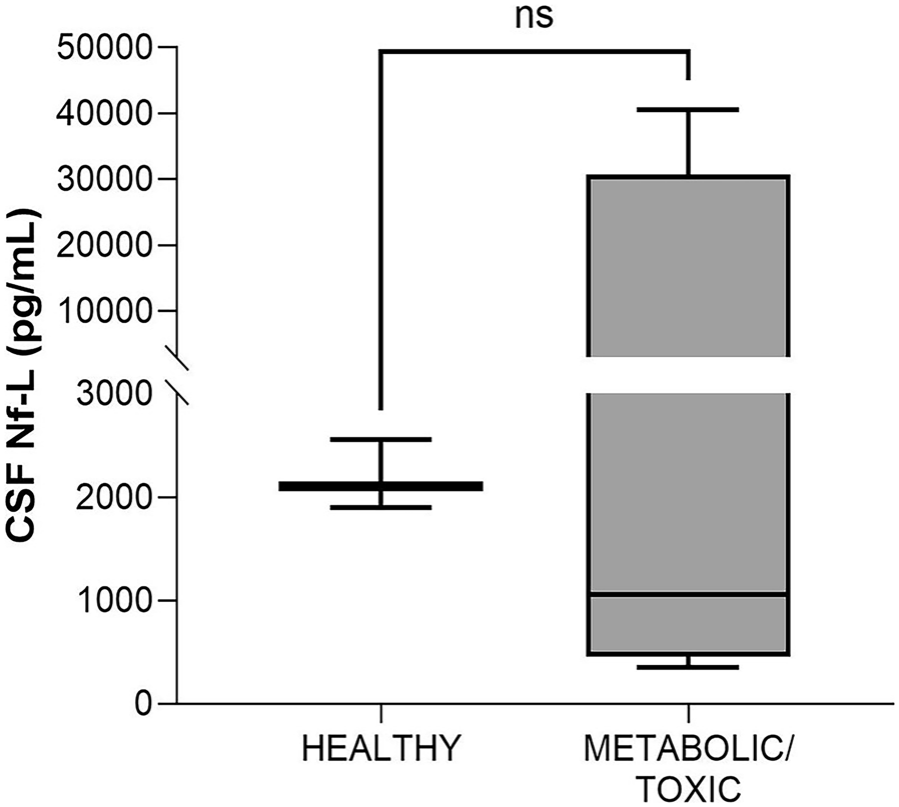
**Comparison of CSF Nf-L levels in healthy cattle and those (age, > 12 years) with CNS and PNS metabolic/toxic disorders.** There was no substantial difference in CSF Nf-L levels between healthy cattle and those with a metabolic/toxic disorder. The median, IQR, maximum and minimum are shown. The Mann–Whitney test was applied. ns denotes not significant. CSF cerebrospinal fluid, IQR interquartile range, Nf-L neurofilament light chain, CNS central nervous system, PNS peripheral nervous system.

A metabolic/toxic disorder was diagnosed in 4 cases: *Clostridium botulinum* (*C. botulinum*) intoxication (*n* = 3) and vitamin B1 deficiency (*n* = 1). There was no significant difference in Nf-L levels between the samples from the healthy and the sick animals (*p* = 0.4, Figure 12).

### Comparison of CSF Nf-L levels in sick cattle

To determine whether Nf-L levels could discern different disease processes, we compared CSF Nf-L concentrations in age-matched sick animals across the VITAMIN D groups.

#### Calves with anomalies vs degenerative disorders vs infectious/inflammatory disorders (age, < 2 months)

**Figure 13:**
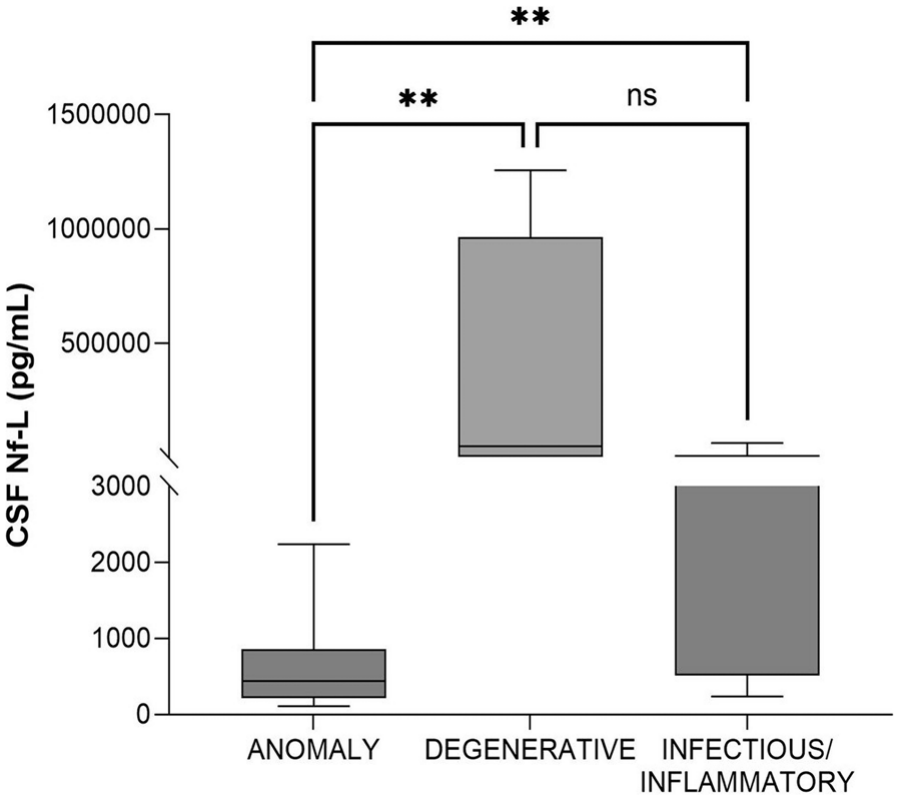
**Comparison between calves (age, < 2 months) with anomaly, degenerative disorder, and infectious/inflammatory disease of the CNS.** The CSF Nf-L concentrations were significantly higher in calves with a neurodegenerative disorder or CNS infection than in those with a CNS anomaly. There were no significant differences in calves with a CNS infection and those with a neurodegenerative disorder. The median, IQR, maximum and minimum are shown. Kruskal–Wallis rank sum test with pairwise comparison was used, and the Benjamini–Hochberg procedure applied to adjust the p-values. Asterisks (*) denote the level of statistical significance: ** p < 0.01. ns denotes not significant. CSF cerebrospinal fluid, CNS central nervous system, IQR interquartile range, Nf-L neurofilament light chain.

CSF Nf-L concentrations were significantly higher in the calves with a neurodegenerative disorder than in those with CNS anomalies (*p* = 0.005) and in the calves with a CNS infection than in those with CNS anomalies (*p* = 0.006). No significant differences were found between the calves with a CNS infection and those with a neurodegenerative disorder (*p* = 0.6, Figure 13).

**Figure 14:**
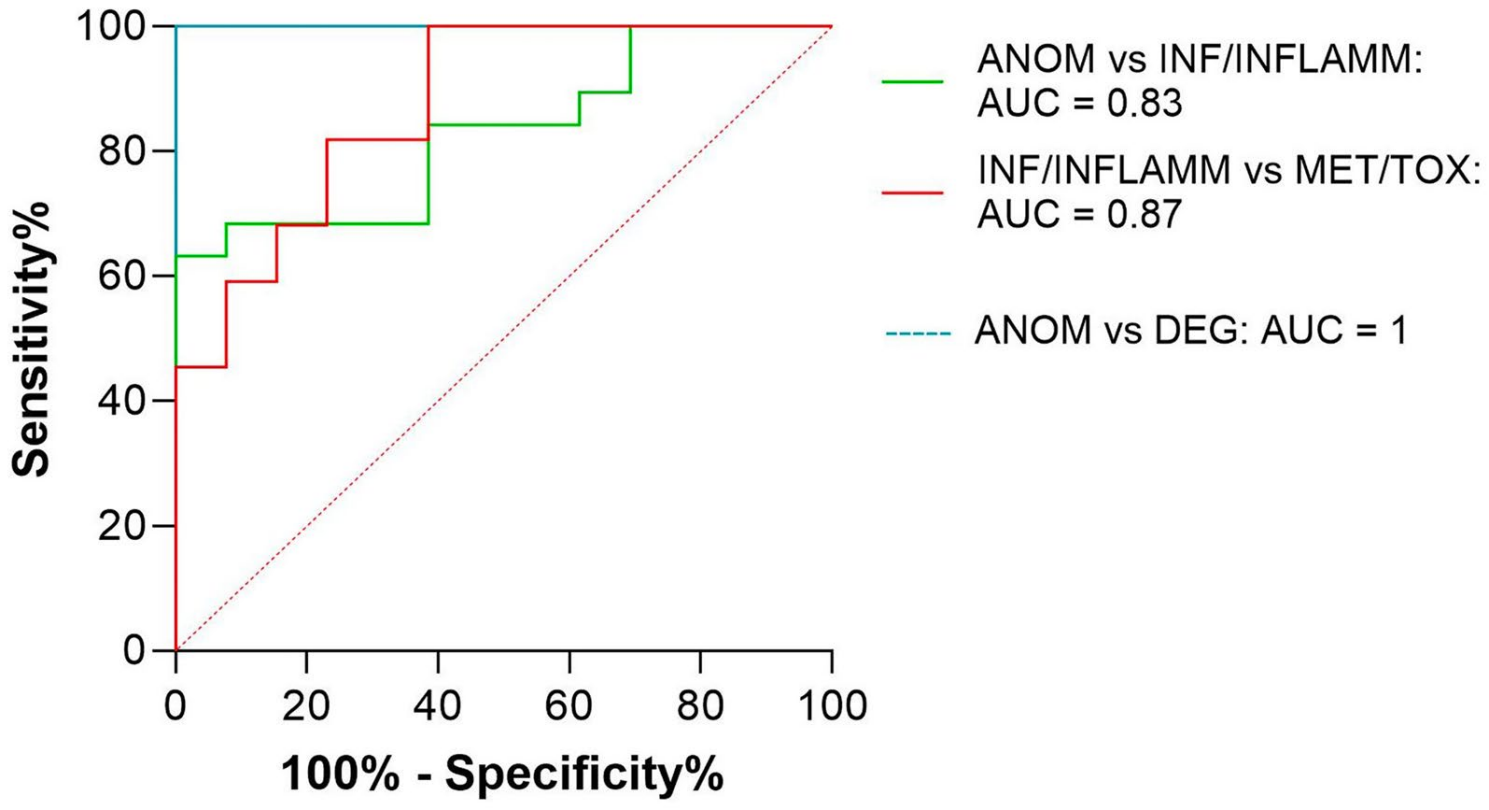
**AUROC curves predicting CNS disorders based on CSF Nf-L concentration.** The AUROC for CSF Nf-L concentration in calves with a CNS anomaly and a neurodegenerative disorder was 1 (light blue line) and the corresponding optimal cut-off to distinguish between them was 3495 pg/mL (Se, 100%, Sp, 100%). The AUROC for CSF Nf-L concentration in calves with a CNS anomaly and with a CNS infection was 0.83 (green line) and the corresponding optimal cut-off to distinguish between them was 467.5 pg/mL (Se, 84.21%, Sp, 61.54%. The AUROC for CSF Nf-L concentration in calves aged 2–12 months with a metabolic/toxic disorder and a CNS infection was 0.87 (red line) and the corresponding optimal cut-off to distinguish between them was 966.5 pg/mL (Se, 68.18%, Sp, 84.62%). The x-axis represents the false positive rate (1-Specificity), and the y-axis represents the true positive rate (Sensitivity). ANOM denotes anomalies, AUROC area under the receiver operating characteristic curve, CSF cerebrospinal fluid, CNS central nervous system, DEG degenerative, INF/INFLAMM infectious/inflammatory, MET/TOX metabolic/toxic, Nf-L neurofilament light chain, Se sensitivity, Sp specificity.

The AUROC was 1 for CSF Nf-L concentration in the calves with a neurodegenerative disorder and CNS anomalies (Figure 14). The corresponding optimal cut-off to distinguish between them was 3495 pg/mL; sensitivity and specificity were 100 and 100%, respectively. The AUROC was 0.83 for CSF Nf-L concentration in the calves with a CNS infection and CNS anomalies (Figure 14). The corresponding optimal cut-off to distinguish between them was 467.5 pg/mL; sensitivity and specificity were 84.21 and 61.54%, respectively.

#### Calves with infectious/inflammatory vs. metabolic/toxic disorders (age, 2–12 months)

**Figure 15:**
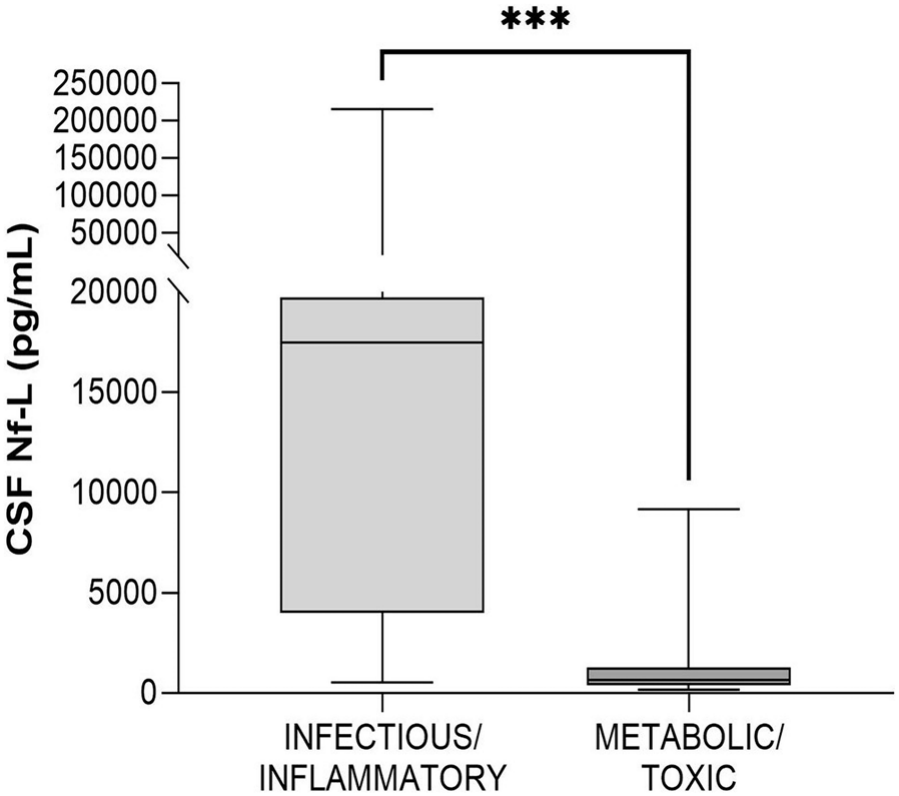
**Comparison between calves (age, 2–12 months) with a CNS infectious/inflammatory disease and a metabolic/toxic disorder.** The CSF Nf-L levels were significantly higher in calves with a CNS infection than in those with a metabolic/toxic disorder. The median, IQR, maximum and minimum are shown. The Mann–Whitney test was used. Asterisks (*) denote the level of statistical significance: *** p < 0.001. CSF cerebrospinal fluid, CNS central nervous system, IQR interquartile range, Nf-L neurofilament light chain.

CSF Nf-L levels were significantly higher in the calves with a CNS infection than in those with a metabolic/toxic disorder (*p* = 0.0001, Figure 15). The AUROC was 0.87 for CSF Nf-L concentration (Figure 14). The corresponding optimal cut-off to distinguish between the two groups of sick animals was 966.5 pg/mL; sensitivity and specificity were 68.18% and 84.62%, respectively.

### Relationship between serum and CSF Nf-L concentration

**Figure 16:**
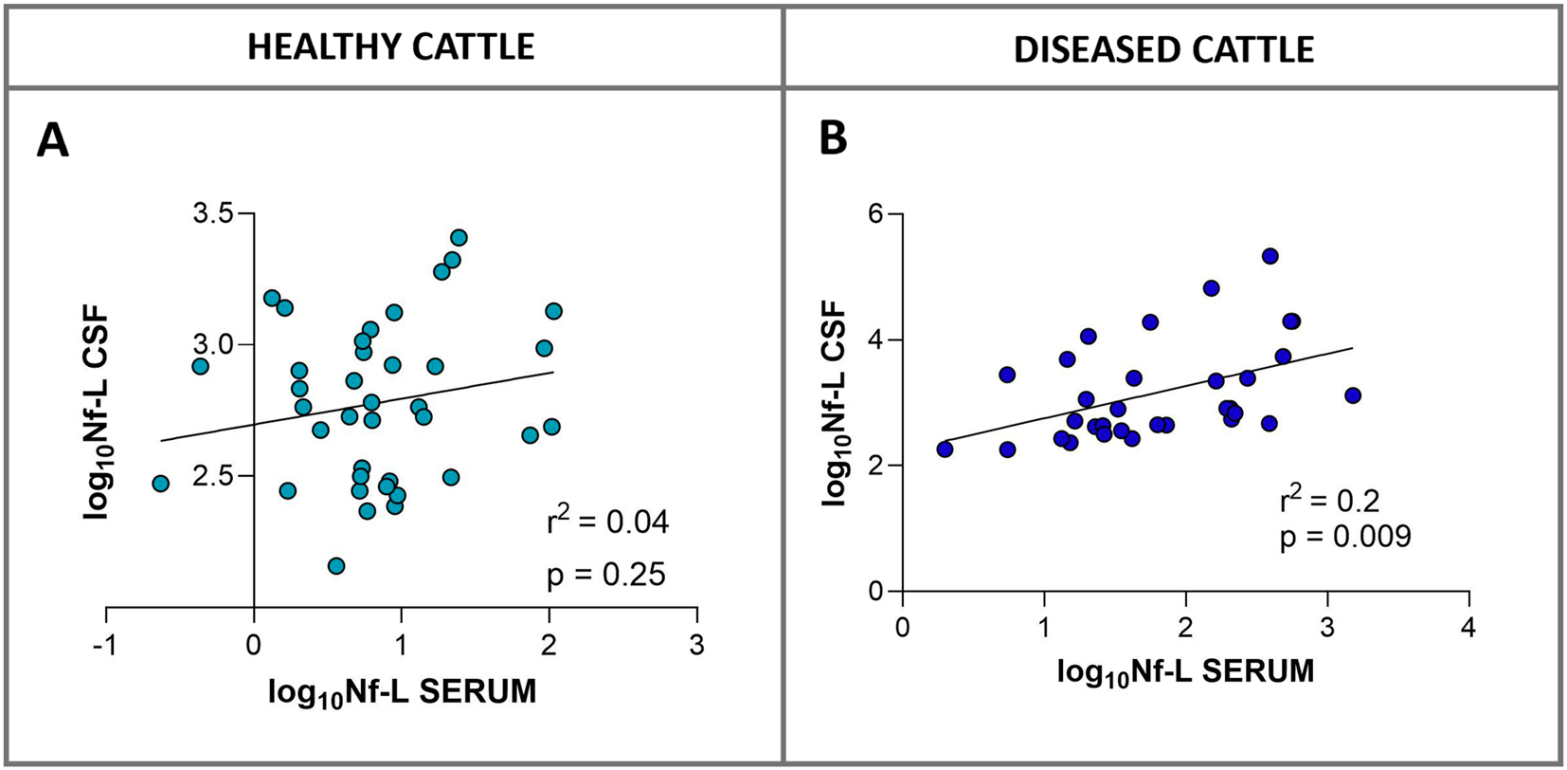
**Relationship between serum and CSF Nf-L concentrations in healthy (A) and sick (B) cattle.** There was no relationship between serum and CSF concentration in the samples from healthy cattle (**A**); there was a weak relationship between serum and CSF Nf-L concentrations in the samples from sick cattle (**B**). The simple linear regression goodness of fit is expressed as r squared (r^2^) and the related *p*-value. The scatter points denote individual data points. CSF cerebrospinal fluid, Nf-L neurofilament light chain.

CSF and serum Nf-L levels were compared in 70 paired samples (38 from healthy and 32 from sick animals) to determine the reliability of using Nf-L measurements from serum samples to predict CSF Nf-L levels. No relationship (r squared = 0.04, *p* = 0.25, Figure 16A) was found between the Nf-L concentrations measured in the samples from the healthy cattle, while a significant albeit weak relationship was found in the samples from the sick cattle (r squared = 0.2, *p* = 0.009, Figure 16B).

## Discussion

Diagnosis of neurological diseases in farm animals is usually made by objective examination and CSF and blood testing of symptomatic animals. Indeed, reaching an etiological diagnosis can be challenging: clinical signs and changes in blood and CSF parameters are often nonspecific, and the feasibility of advanced diagnostic imaging in large animals is limited by both the considerable costs involved and the patient’s size [34]. There is a growing need among veterinarians for an intra-life diagnostic test that can improve the accuracy of differential diagnosis of neurological signs and that is sensitive, reliable, minimally invasive, and inexpensive.

The Ella microfluidic immunoassay is a new commercially available platform commonly used on human samples to quantify soluble biomarkers such as cytokines, chemokines, and Nf-L. Different from other quantification systems that analyze a few samples in the same run, this platform can measure 72 samples simultaneously. Moreover, the samples are uploaded in a cartridge and the microfluidic system splits each sample into three different microchannels to create a triplicate, thus reducing operator errors. The system returns results in less than 90 min and produces quantitative and reproducible data from just 25 µL of sample. While Ella and all the other commercially available equivalent systems are certified for use on human samples, we found no information about its applicability on animal samples. To fill this gap, ours is the first study to report data on Nf-L quantification in CSF and serum samples from cattle.

### Nf-L stability at two different storage temperatures

Prior to January 2021, CSF samples from bovine patients referred to the VTH were stored at −20 °C. Our primary objective was to determine the viability of these samples and assess the long-term stability of Nf-L at this temperature. Previous studies on samples of human CSF and serum showed that Nf-L concentrations are unaffected by storage at −20 °C for 21 days [35, 36]. Additionally, storage of CSF samples at the reference temperature of −80 °C for up to 11 years reportedly had no significant impact on Nf-L concentration [37]. Here we compared samples prospectively enrolled and stored both at −20 °C and −80 °C and found a strong correlation between them, indicating that storing samples at −20 °C for extended periods has no effect on Nf-L stability. Therefore, these samples were included in the analysis.

### Nf-L concentrations in healthy cattle

Here we report for the first time reference values for normal serum and CSF Nf-L levels in a population of healthy cattle. We also looked for an association between animal age and Nf-L concentrations, as previously reported in healthy humans and dogs [38, 39]. Indeed, under normal physiological conditions, axons consistently release low levels of Nf-L into the CSF and then into the bloodstream in a widely recognized correlation with age [17, 38, 40]. We observed a significant positive relationship between Nf-L levels and age in this study population of healthy adult cattle, which could be primarily attributed to the degeneration of neurons, changes in axon structure, and metabolic alterations associated with aging as described in humans [41, 42]. In calves, however, there was a weak predictive role of age for Nf-L concentrations in the serum samples. According to the human literature on healthy children, we would expect a negative correlation between Nf-L levels and age, which mirrors the phenomenon of synapse pruning and remodeling during normal neuron development and accelerates in the first years after birth, before stabilizing as growth progresses [40, 43]. In their study involving a group of healthy dogs categorized as puppy/junior, adult/mature, and senior/geriatric dogs, Panek and colleagues found that plasma Nf-L concentrations had a significant positive correlation with age but no difference between young and old dogs was found [17]. Our findings in the samples from healthy calves suggest a potentially negative correlation between age and Nf-L concentration. However, the patients in this group predominantly fell into two age subgroups: those aged < 2 months and those aged 5–7 months. This asymmetric distribution may have influenced our results, making it challenging to establish a clear association between age and Nf-L levels in young animals.

### Comparison of CSF Nf-L concentration in healthy and sick cattle

When we compared CSF Nf-L concentration between healthy and sick cattle, we found significantly higher CSF Nf-L levels in those with CNS degenerative and infectious conditions than in the healthy controls. However, no differences in CSF Nf-L levels were found between the samples from the healthy and from the cases of CNS anomalies or metabolic/toxic disorders. Differences in Nf-L levels are known in human medicine, as Nf-L concentrations have been observed to increase in both CSF and blood following axonal injury or degeneration triggered by a spectrum of CNS and peripheral nervous system (PNS) diseases [5]. Furthermore, CSF Nf-L cut-off values have been established to help differentiate healthy cattle from those affected by degenerative or infectious conditions. However, because Nf-L levels are not specific to any single disease, these cut-off values should be interpreted cautiously. For accurate diagnosis, Nf-L measurements should always be considered alongside the animal's medical history and a comprehensive clinical examination.

Notably, in this study some etiopathological subgroups included relatively few subjects. This limitation is due to the rarity of certain neurological disorders in large animal neurology, which restricted the ability to enroll larger numbers in each subgroup.

#### Healthy vs calves with degenerative disorders

Neurodegenerative conditions are rare in large animals. All the calves in the neurodegenerative group were from the same herd and were diagnosed with spinal muscular atrophy (SMA), a severe inherited disorder characterized by the degeneration of motor neurons and resulting in muscle weakness. Human studies have shown a diagnostic role for Nf-L levels in children with SMA [44, 45]. These observations are shared by our findings for the relevance of Nf-L in diagnosing SMA in cattle. This holds particular relevance when we consider that physicochemical analysis of the CSF, the primary method for diagnosing ante-mortem CNS diseases in cattle, is unremarkable in neurodegenerative diseases. This suggests the potential use of Nf-L quantification in the diagnosis of neurodegenerative disorders in cattle, although this finding should be validated with a larger cohort of cattle affected by various neurodegenerative disorders.

#### Healthy vs cattle with infectious/inflammatory disorders

The veterinary literature describes the susceptibility of large animals to etiological agents based on age. For example, *Listeria monocytogenes* is most prevalent in adult ruminants, while *Escherichia coli*, *Salmonella sp*., and *Pasteurella sp.* are common in calves [46]. We thought it reasonable to group the animals with a CNS infection by age for our analyses. The CSF Nf-L levels were significantly higher in the groups of sick animals. Moreover, we identified at post-mortem examination a subset of calves with confirmed *E. coli* CNS infections. This enabled us to conduct further analysis, in which we compared the sick and the healthy calves and found the difference to be statistically significant, suggesting the potential of CSF Nf-L in discerning between the underlying causative pathogen of CNS infection. In human medicine, controversy surrounds the use of Nf-L concentration to discriminate between causes of suspected CNS infections. A recent study reported higher CSF Nf-L levels in patients with pneumococcal meningitis than in those with non-pneumococcal meningitis [6]. Unfortunately, we were unable to identify the underlying pathogen in the majority of cases within our population, thus confirming the diagnostic limitations of standard ante-mortem examination as reported previously [47, 48]. Future studies involving animals with identified causative pathogens may provide valuable insights into whether Nf-L levels can aid in distinguishing between the various microorganisms responsible for CNS infections in cattle.

#### Healthy vs calves with anomalies

As expected, there was no difference in CSF Nf-L concentration between healthy and sick calves with CNS anomalies. This is not surprising, considering that active ongoing axonal damage is not expected in the course of CNS anomalies.

#### Healthy vs cattle with metabolic/toxic disorders

Common metabolic disorders of the CNS in cattle are often attributed to nutritional deficiencies, including electrolytes imbalances in calcium (Ca^2+^) and magnesium (Mg^2+^), thiamine and vitamin A deficiency, with a predisposition in relation to age [33]. We grouped the sick cattle by age and conducted a separate analysis on each group. Since we found no differences in CSF Nf-L concentration between the healthy and the sick animals, we then performed separate analyses of samples from three subgroups of cattle with different metabolic conditions: hypocalcemia/hypomagnesemia, vitamin A deficiency, and thiamine deficiency. CSF Nf-L levels were significantly higher in the cattle with thiamine deficiency, which can lead to necrosis of the cerebral cortex. This structural CNS lesion, which is not observed in the animals with other metabolic disorders, is a likely explanation for this observation. Thiamine deficiency typically presents with unremarkable CSF findings, necessitating diagnosis by expensive and laborious testing [33]. Our results suggest the measurement of Nf-L for early diagnosis of thiamine deficiency. Early diagnosis and prompt treatment may resolve the clinical signs or lead to recovery with mild deficits that do not impact on an animal’s breeding potential [49].

Finally, no significant differences were found in Nf-L levels between the healthy cattle and those diagnosed with botulism. This may be because *C. botulinum* neurotoxin inhibits the neurotransmitter release [33] and does not induce axonal damage.

### Comparison of CSF Nf-L levels in sick cattle

CSF Nf-L measurement was useful in differentiating calves with a CNS anomaly from those with SMA. In particular, Nf-L levels exceeding 3495 pg/mL strongly indicate SMA, with both sensitivity and specificity reaching 100%. This is particularly important, given that both disorders often present similar signalment and are extremely challenging to diagnose ante-mortem. CSF analysis is unremarkable in both. CNS anomalies require advanced diagnostic imaging procedures to obtain an accurate ante-mortem description of the suspected malformation. These procedures are rarely performed in large animals because they are expensive and require general anesthesia [34]. Differently, SMA has been linked to a genetic mutation of the FVT1 gene across various cattle breeds and diagnosis involves expensive genetic tests [50]. Due to these limitations, definitive diagnosis is usually made post-mortem. We suggest that, in presence of clinical suspects, Nf-L measurement can provide a crucial ante-mortem tool to differentiate between these conditions.

Measuring Nf-L levels in CSF may also help distinguish between calves with CNS anomalies and those with CNS infections, as Nf-L levels above 467.5 pg/mL indicate a likely CNS infection. These disorders often present similar signalment and clinical signs, particularly in cases where malformations are not clinically evident. CNS infections can usually be distinguished by an abnormal increase in protein concentration and total and differential nucleated cell counts in the CSF [48]. Nf-L measurement may provide an additional tool to reinforce the diagnosis and confirm the result of CSF analysis.

Finally, metabolic disorders can be distinguished from CNS infections in juvenile cattle by their CSF Nf-L levels. Specifically, Nf-L levels exceeding 966.5 pg/mL are indicative of CNS infections. Metabolic disorders and CNS infections typically shared similar clinical symptoms, such as disorientation, tremor, hyperesthesia, opisthotonus, convulsions, and recumbency [33, 49]. A prompt ante-mortem diagnosis is key to the choice of appropriate treatment, to reduce mortality rate and inappropriate use of antibiotics. In this context, CSF Nf-L concentration showed potential in the differential diagnosis of these disorders.

The results indicate that Nf-L quantification could be a valuable tool for differentiating neurological disorders with nonspecific symptoms in field settings. However, similar to its use in human medicine, Nf-L measurement primarily reflects neuronal damage across the CNS and PNS rather than pointing to a specific disease [51]. Consequently, while Nf-L levels provide useful insights, they should always be interpreted alongside the patient’s clinical history and comprehensive evaluation in a clinical setting.

### Relationship between serum and CSF Nf-L concentration

We found a weak role of serum Nf-L in predicting CSF Nf-L levels in sick cattle, and no relationship between CSF and serum Nf-L levels in healthy cattle. A meta-analysis of CSF and blood Nf-L in human patients reported a moderate correlation, with a pooled correlation coefficient estimate for CSF and blood Nf-L (r = 0.72) [52]. A potential explanation for our findings may be the inclusion of animals with minor or no impairment of the blood–brain barrier (BBB). The BBB is a semipermeable complex that separates the CSF from the blood and protects the CNS. Neuroinflammation and neurodegeneration can cause dysfunction of the BBB, making it hyperpermeable and enabling a broader range of molecules to pass across it [53]. Future studies involving a larger cohort of sick cattle grouped by etiological diagnosis are needed to strengthen our findings.

To date, no other studies in veterinary medicine have explored the feasibility of utilizing serum Nf-L concentration as a surrogate measure for CSF Nf-L. This gap is important, as CSF collection is invasive, unlike an ideal biomarker, that is minimally invasive, sensitive, reliable, and inexpensive.

The present study has several limitations. The sample of healthy animals was not numerous enough for an accurate description of the reference physiological range of Nf-L concentration in CSF and serum. Studies in healthy cattle are therefore warranted to better explore the relationship with age. Potential confounding variables that might influence Nf-L levels were not fully explored in this study. In human medicine, factors such as body mass index (BMI), renal function, and total blood volume have been shown to impact Nf-L measurements; however, age is recognized as the primary factor affecting Nf-L concentrations [52, 53]. Given the challenges of collecting detailed physiological and nutritional information for cattle in field conditions and the partial retrospective nature of the study, age was the only confounding factor considered for Nf-L levels. Given the relatively low prevalence of certain disorders, particularly neurodegenerative ones, our cohort of sick animals did not encompass all neurological disorders occurring in cattle. Further research is needed to expand our results. The relationship between serum and CSF Nf-L levels was weak, owing to the inclusion of few sick animals in the analysis. Future studies may help to strengthen this association.

In conclusion, the present study suggests that Nf-L measurement may provide a reliable biomarker to improve the accuracy of differential diagnosis in cattle with neurological disorders. Furthermore, our results suggest that Nf-L is extremely stable at different storage conditions, easily detectable in both serum and CSF, and may aid in ante-mortem etiopathological diagnosis in cattle. This is a crucial point in cattle management, as neurological disorders are responsible for economic losses, mortality, and diminished productivity. Taken together, our findings offer a starting point for future studies to better characterize Nf-L levels in diseases of the CNS and the PNS in cattle and its potential role in discriminating among neurological disorders.

## Supplementary Information

**Additional file 1: Demographics of healthy cattle grouped by age.** Data are expressed as median and interquartile range (IQR) for continuous variables and as absolute frequency and percentage for categorical variables.

**Additional file 2: Demographics of sick cattle grouped by age.** Etiological diagnosis is expressed according to the VITAMIN D acronym. Data are expressed as median and interquartile range (IQR) for continuous variables and as absolute frequency and percentage for categorical variables.

**Additional file 3: CSF Nf-L concentration in healthy cattle grouped by age.** CSF denotes cerebrospinal fluid, Nf-L neurofilament light chain, Q1 first quartile, Q3 third quartile.

**Additional file 4: CSF Nf-L concentration in sick cattle grouped by age-and the VITAMIN D acronym.** CSF denotes cerebrospinal fluid, Nf-L neurofilament light chain, Q1 first quartile, Q3 third quartile.

## Data Availability

All data generated or analyzed during this study are included in this published article and its supplementary information files.
