# Supplementary material for: Neurofilament light chain (Nf-L) in cerebrospinal fluid and serum as a potential biomarker in the differential diagnosis of neurological diseases in cattle
Source: Vet Res. 2025 Jan 10;56:6. doi: 10.1186/s13567-024-01441-4 (PMC11724550; doi:10.1186/s13567-024-01441-4)
Supplement: Supplementary file 1 — Additional file 1: Demographics of healthy cattle grouped by age. Data are expressed as median and interquartile range (IQR) for continuous variables and as absolute frequency and percentage for categorical variables. [file 13567_2024_1441_MOESM1_ESM.docx]

**Demographics of healthy cattle grouped by age.** Data are expressed as median and interquartile range (IQR) for continuous variables and as absolute frequency and percentage for categorical variables.

| HEALTHY ANIMALS | | | | | |
| --- | --- | --- | --- | --- | --- |
| GROUP | AGE | BODY WEIGHT -kg | SEX (no., %) | BREED (no., %) |  |
| < 2 mths (*n* = 8) | 9.5 days (8-16.3) | 52.5 (38.75-62.5) | Male (6; 75)  Female (2; 25) | Piedmontese (6; 75)  Holstein (2; 25) |  |
| ≥ 2-12 mths (*n* = 26) | 5.2 mths (5.1-6.9) | 193 (190-197) | Male (18; 69.2)  Female 8; 30.8) | Holstein (26; 100) |  |
| ≥ 1-6 years (*n* = 6) | 37.4 mths (23-62) | 560 (482.5.577.5) | Female (*n* = 6; 100) | Piedmontese 6; 100) |  |
| ≥ 6-12 years (*n* = 6) | 99.9 mths (79.8-119) | 635 (620-672.5) | Female (6; 100) | Piedmontese (5; 83.3)  Holstein (1; 16.7) |  |
| ≥ 12 years (*n* = 3) | 180 mths (164.5-181.3) | 720 (710-735) | Female (3; 100) | Piedmontese (3; 100) |  |
