# Supplementary material for: Neurofilament light chain (Nf-L) in cerebrospinal fluid and serum as a potential biomarker in the differential diagnosis of neurological diseases in cattle
Source: Vet Res. 2025 Jan 10;56:6. doi: 10.1186/s13567-024-01441-4 (PMC11724550; doi:10.1186/s13567-024-01441-4)
Supplement: Supplementary file 2 — Additional file 2: Demographics of sick cattle grouped by age. Etiological diagnosis is expressed according to the VITAMIN D acronym. Data are expressed as median and interquartile range (IQR) for continuous variables and as absolute frequency and percentage for categorical variables. [file 13567_2024_1441_MOESM2_ESM.docx]

**Demographics of sick cattle grouped by age. Etiological diagnosis is expressed according to the VITAMIN D acronym.** Data are expressed as median and interquartile range (IQR) for continuous variables and as absolute frequency and percentage for categorical variables.

| SICK ANIMALS | | | | |
| --- | --- | --- | --- | --- |
| GROUP | AGE | BODY WEIGHT - kg | SEX (no., %) | BREED (no., %) |
| ANOMALY  < 2 mths (*n* = 13) | 7 days  (3-10) | 40 (30-50) | Male (8; 61.5)  Female (5; 38.5) | Piedmontese (12; 92.3)  Mixed (1; 7.7) |
| DEGENERATIVE  < 2 mths (*n* = 4) | 1 mth (1-1) | 42.5(40-46.25) | Male 4; 100) | Blonde D’Aquitaine  (4; 100) |
| INFECTIOUS/ INFLAMMATORY  <2 mths (*n* = 19) | 8 days (5-19.5) | 42.5 (40-50) | Male (14; 73.7)  Female (5; 26.3) | Piedmontese 16; 84.2)  Mixed (2; 10.5)  Holstein (1; 5.3) |
| INFECTIOUS/ INFLAMMATORY  ≥ 2-12 mths (*n* = 13) | 6 mths (5-8) | 350 (270-450) | Male (5; 38.5)  Female (8; 61.5) | Piedmontese (8; 61.5)  Holstein (2; 15.4)  Limousine (1; 7.7)  Blonde D’Aquitaine  (1; 7.7)  Valdostana pezzata rossa  (1; 7.7) |
| INFECTIOUS/  INFLAMMATORY  ≥ 1-6 years (*n* = 7) | 24.3 mths (17.7-42.6) | 650 (610-650) | Male (1; 14.3)  Female (6; 85.7) | Piedmontese (5; 71.4)  Holstein (1; 14.3)  Limousine (1; 14.3) |
| METABOLIC/ TOXIC  ≥ 2-12 mths (*n* = 15) | 3,6 mths (3-7) | 180 (150-450) | Male (8; 53.3)  Female (7; 46.7) | Piedmontese (13; 86.7)  Holstein (2; 13.3) |
| METABOLIC/ TOXIC  ≥ 12 years (*n* = 4) | 170.3 mths (164.4-170.3) | 585 (537.5-640) | Female (4; 100) | Piedmontese (4; 100) |
