# Supplementary material for: Neurofilament light chain (Nf-L) in cerebrospinal fluid and serum as a potential biomarker in the differential diagnosis of neurological diseases in cattle
Source: Vet Res. 2025 Jan 10;56:6. doi: 10.1186/s13567-024-01441-4 (PMC11724550; doi:10.1186/s13567-024-01441-4)
Supplement: Supplementary file 3 — Additional file 3: CSF Nf-L concentration in healthy cattle grouped by age. CSF denotes cerebrospinal fluid, Nf-L neurofilament light chain, Q1 first quartile, Q3 third quartile. [file 13567_2024_1441_MOESM3_ESM.docx]

**CSF Nf-L concentration in healthy cattle grouped by age.** CSF denotes cerebrospinal fluid, Nf-L neurofilament light chain, Q1 first quartile, Q3 third quartile.

| HEALTHY ANIMALS | | | | | |
| --- | --- | --- | --- | --- | --- |
| GROUP | CSF Nf-L (pg/mL) | | | | |
|  | MEDIAN | Q1 | Q3 | MINIMUM | MAXIMUM |
| < 2 mths  (*n* = 8) | 761.5 | 354.5 | 1251 | 64 | 3531 |
| ≥ 2-12 mths  (*n* = 26) | 361.5 | 278 | 544.8 | 144 | 1506 |
| ≥ 1-6 years  (*n* = 6) | 688 | 516.8 | 854.3 | 474 | 936 |
| ≥ 6-12 years  (*n* = 6) | 931.5 | 652.5 | 1202 | 567 | 1383 |
| ≥ 12 years  (*n* = 3) | 2105 | 1898 | 2558 | 1898 | 2558 |
