# Supplementary material for: Neurofilament light chain (Nf-L) in cerebrospinal fluid and serum as a potential biomarker in the differential diagnosis of neurological diseases in cattle
Source: Vet Res. 2025 Jan 10;56:6. doi: 10.1186/s13567-024-01441-4 (PMC11724550; doi:10.1186/s13567-024-01441-4)
Supplement: Supplementary file 4 — Additional file 4: CSF Nf-L concentration in sick cattle grouped by age-and the VITAMIN D acronym. CSF denotes cerebrospinal fluid, Nf-L neurofilament light chain, Q1 first quartile, Q3 third quartile. [file 13567_2024_1441_MOESM4_ESM.docx]

**CSF Nf-L concentration in sick cattle grouped by age and the VITAMIN D acronym.** CSF denotes cerebrospinal fluid, Nf-L neurofilament light chain, Q1 first quartile, Q3 third quartile.

| SICK ANIMALS | | | | | | |
| --- | --- | --- | --- | --- | --- | --- |
| GROUP | CSF Nf-L (pg/mL) | | | | |  |
|  | MEDIAN | Q1 | Q3 | MINIMUM | MAXIMUM |  |
| ANOMALY  < 2 mths  (*n* = 13) | 442 | 218.5 | 856 | 108 | 2238 |  |
| DEGENERATIVE < 2 mths  (*n* = 4) | 49971 | 5216 | 965636 | 4751 | 1256404 |  |
| INFECTIOUS/ INFLAMMATORY < 2 mths  (*n* = 19) | 8863 | 513 | 12821 | 238 | 66326 |  |
| INFECTIOUS/ INFLAMMATORY  ≥ 2-12 mths  (*n* = 13) | 17474 | 3987 | 19750 | 513 | 215740 |  |
| INFECTIOUS/  INFLAMMATORY  ≥ 1-6 years  (*n* = 7) | 3546 | 1900 | 12190 | 1279 | 120195 |  |
| METABOLIC/ TOXIC  ≥ 2-12 mths  (*n* = 15) | 449 | 318 | 1253 | 180 | 9174 |  |
| METABOLIC/ TOXIC  ≥ 12 years  (n = 4) | 1061 | 457.5 | 30780 | 350 | 40593 |  |
